# Assessment of Serum Pepsinogens with and without Co-Testing with Gastrin-17 in Gastric Cancer Risk Assessment—Results from the GISTAR Pilot Study

**DOI:** 10.3390/diagnostics12071746

**Published:** 2022-07-19

**Authors:** Claudia Robles, Dace Rudzite, Inese Polaka, Olga Sjomina, Lilian Tzivian, Ilze Kikuste, Ivars Tolmanis, Aigars Vanags, Sergejs Isajevs, Inta Liepniece-Karele, Danute Razuka-Ebela, Sergej Parshutin, Raul Murillo, Rolando Herrero, Jin Young Park, Marcis Leja

**Affiliations:** 1 Early Detection, Prevention and Infections Branch, International Agency for Research on Cancer, 69372 Lyon, France; rmurillo@husi.org.co (R.M.); rherrero@acibcr.com (R.H.); parkjy@iarc.fr (J.Y.P.); 2 Cancer Epidemiology Research Program, Catalan Institute of Oncology, IDIBELL, 08908 L’Hospitalet de Llobregat, Spain; 3 Institute of Clinical and Preventive Medicine, Faculty of Medicine, University of Latvia, 1007 Riga, Latvia; dacerudzite2008@inbox.lv (D.R.); inese.polaka@lu.lv (I.P.); olga_sjomina@inbox.lv (O.S.); liliana.tz@gmail.com (L.T.); ikikuste@gmail.com (I.K.); sergisajevs@inbox.lv (S.I.); intaliepniecekarele@inbox.lv (I.L.-K.); d.razuka.ebela@gmail.com (D.R.-E.); sergejs.parsutins@lu.lv (S.P.); marcis.leja@lu.lv (M.L.); 4 Department of Research, Riga East University Hospital, 1038 Riga, Latvia; 5 Digestive Diseases Centre GASTRO, 1586 Riga, Latvia; ivars.tolmanis@gastrocentrs.lv (I.T.); mako@inbox.lv (A.V.); 6 Academic Histology Laboratory, 1073 Riga, Latvia; 7 Centro Javeriano de Oncología, Hospital Universitario San Ignacio, Bogotá 11001, Colombia; 8 Agencia Costarricense de Investigaciones Biomedicas, Fundacion INCIENSA, San Jose 2250, Costa Rica

**Keywords:** serum pepsinogens, gastrin-17, gastric cancer prevention, screening, public health

## Abstract

Introduction––Serum pepsinogen tests for gastric cancer screening have been debated for decades. We assessed the performance of two pepsinogen assays with or without gastrin-17 for the detection of different precancerous lesions alone or as a composite endpoint in a Latvian cohort. Methods––Within the intervention arm of the GISTAR population-based study, participants with abnormal pepsinogen values by ELISA or latex-agglutination tests, or abnormal gastrin-17 by ELISA and a subset of subjects with all normal biomarker values were referred for upper endoscopy with biopsies. Performance of biomarkers, corrected by verification bias, to detect five composite outcomes based on atrophy, intestinal metaplasia, dysplasia or cancer was explored. Results––Data from 1045 subjects were analysed, of those 273 with normal biomarker results. Both pepsinogen assays showed high specificity (>93%) but poor sensitivity (range: 18.4–31.1%) that slightly improved when lesions were restricted to corpus location (40.5%) but decreased when dysplasia and prevalent cancer cases were included (23.8%). Adding gastrin-17 detection, sensitivity reached 33–45% while specificity decreased (range: 61.1–62%) and referral rate for upper endoscopy increased to 38.6%. Conclusions––Low sensitivity of pepsinogen assays is a limiting factor for their use in population-based primary gastric cancer screening, however their high specificity could be useful for triage.

## 1. Introduction

In many countries, gastric cancer incidence rates have been steadily decreasing over the last decades by changes in social determinants without major intervention of health services [1], but despite this declining trend it is the third cancer cause of death in men [2]. However, the observed social inequalities due to its higher incidence in many developing countries and its expected increase in worldwide burden due to demographic changes highlight the need to identify potential prevention strategies.

Symptomatic gastric cancer is most frequently diagnosed at an advanced stage when a complete cure is not possible [3]. Therefore, it is important to diagnose it between the onset and the appearance of symptoms [4], or during the period of advanced precancerous lesions when patients could be under surveillance [5].

Early detection of gastric cancer by endoscopy is a costly and invasive test. Therefore, a pre-selection of population at higher risk could substantially reduce costs due to a lower number of subjects undergoing endoscopy and the achievement of higher participation rates due to a higher acceptance based on a previous positive test result.

Atrophic gastritis and intestinal metaplasia (IM) are well-known gastric precancerous conditions that can be triggered by a *Helicobacter pylori* (*H. pylori*) infection [6]. Staging of these conditions using the operative link for gastritis assessment (OLGA) and the operative link for gastric IM (OLGIM) scoring systems [7] has proven useful for gastric cancer risk stratification in research settings [8].

Gastric atrophy causes a reduction in functional gland cells and their production of enzymes. Atrophy in the corpus causes a reduction in pepsinogen I (PgI) levels and the PgI/PgII ratio, whereas in the antrum it reduces gastrin 17 (G-17) levels [9]. Due to the correlation with gastric levels, detection of serum pepsinogens is considered the most useful non-invasive test to explore the status of the gastric mucosa [10].

However, whether pepsinogens can be used for gastric cancer screening is under debate [10,11]. Several meta-analyses, reviewed by Bang et al. [12], have summarised the use of serum pepsinogens for the early detection of gastric atrophy or cancer, reporting moderate diagnostic accuracy. However, these studies show some limitations; (1) most pepsinogen studies were conducted in Asian countries, raising concerns about the generalizability of the results to non-Asian populations, (2) the diagnostic accuracy is largely dependent on the gastric atrophy definition as well as the technology and positivity cut-offs used to measure pepsinogens [13], (3) concerns have been raised about translating results between the different methods available for pepsinogen assessment (enzyme-linked immunosorbent -ELISA- and latex-agglutination assays) [14], and (4) available studies have evaluated the performance of pepsinogen testing for atrophy or gastric cancer independently, but in practice, screening programs should consider risk of atrophy, cancer precursors and invasive cancer altogether.

On the other hand, the limited sensitivity of serum pepsinogens to detect antrum-restricted atrophy has led to the potential strategy of combining pepsinogen testing to gastrin-17 testing [15] or to *H. pylori* testing [16].

In the present study we assessed the performance of two different pepsinogen detection assays in plasma (an ELISA and a latex-agglutination assay) in detection of gastric precancerous lesions alone or as a composite endpoint to assess its potential use in gastric cancer screening. We addressed the possibility of improving performance by evaluating different cut-off values as well by adding the detection of gastrin-17 assay to pepsinogens. The study was performed within the population-based GISTAR Pilot study conducted in Latvia.

## 2. Materials and Methods

### 2.1. Study Population

The GISTAR study [17] is an ongoing study aiming to determine the efficacy of *H. pylori* eradication combined with pepsinogen testing to reduce gastric cancer mortality. Prior to the main GISTAR study, a pilot study was conducted to assess different serologic screening options for further assessment of cancer incidence within the trial as well as to test the procedures, tools and infrastructures for subsequent use in the trial.

Between October 2013 and December 2015, 1724 men and women aged 40–65 years living in Cēsis, Alūksne, Ludza and Saldus regions of Latvia were randomly allocated to the intervention arm of the GISTAR Pilot study [18]. Potential participants were identified using general practitioners’ registries and invited to participate via phone calls or letters. Exclusion criteria included: personal history of gastric cancer diagnosis or gastric resections due to benign disease except for ulcer suturing or vagotomy, treatment for *H. pylori* eradication within the previous 12 months, presence of alarm symptoms for digestive or other disease, pathological findings suggestive of serious disease requiring immediate management, and other physical or mental conditions that might impair participation or protocol compliance.

### 2.2. Serum Biomarkers and H. pylori Testing Used during the Pilot Study

Study participants were screened for *H. pylori* infection and serum levels of pepsinogen and gastrin-17 biomarkers were determined under fasting conditions. Specifically, serum pepsinogen biomarkers (PgI and PgII) were measured using both an enzyme-linked immunosorbent assay (ELISA) test by Biohit Plc. (Helsinki, Finland) and latex-agglutination test by Eiken Chemical Co. (Tokyo, Japan) whereas serum gastrin-17 levels were measured using an ELISA by Biohit Plc. (Helsinki, Finland). Subjects that met any of the following predefined criteria: ELISA PgI/PgII ratio < 3; latex-agglutination PgI/PgII ratio < 3 and Pgl < 70 ng/mL; or G-17 < 1 pmol/L) were referred for upper endoscopy. A random selection of subjects with normal results in all biomarkers from different blood processing batches were also invited to get a voluntary endoscopy exploration.

*H. pylori* infection was determined by IgG antibody levels against *H. pylori* > 30 EIU using ELISA (Biohit Plc.).

## 3. Biopsy Sampling and Histopathological Assessment

All upper endoscopies procedures were standardized. A conventional white light endoscopy was used, except in specific cases in which an chromoendoscopy was used based on clinical judgement. Five non-targeted biopsies from the corpus and antrum (both greater and lesser curvature) as well as incisura angularis (lesser curvature) according to the updated Sydney system [19]. Additional gastric targeted biopsies were obtained from 33 subjects. Gastric biopsies were fixed in 10% buffered formalin, processed and stained with haematoxylin and eosin, modified Giemsa as well as Alcian blue as routine at the Academic Histology Laboratory in Riga. Histopathological assessment was performed by two independent expert pathologists (SI, ILK) and reported according to the updated Sydney System [19] as well as the operative link for gastritis assessment (OLGA) and operative link for gastric IM (OLGIM) scoring systems [7]. By considering better interobserver agreement for IM than atrophy detection [20,21], the presence of atrophy was considered only if moderate-to-severe atrophy was reported, however, in case of IM, mild lesions were also considered. Dysplasia was graded as indefinite, low or high grade according to the Vienna classification [22].

The targeted conditions or outcomes explored included: (a) *severe atrophy* (equivalent to OLGA Stage 3 or more; OLGA ≥ 3), (b) *severe IM* (equivalent to OLGIM Stage 3 or more; OLGIM ≥ 3) and (c) a composite endpoint of *high risk lesions* including OLGA ≥3, OLGIM ≥ 3, any type of dysplasia (*n* = 82) or cancer (*n* = 3).

Since the presence of any precancerous lesions restricted to the corpus, contrary to most isolated antrum lesions, require surveillance [5] we also explored (d) corpus-restricted *severe atrophy and/or intestinal metaplasia* and (e) a composite outcome of corpus-restricted *severe atrophy and/or intestinal metaplasia including dysplasia or cancer irrespective of its location*.

### Statistical Analysis

In the present study we explored the performance of the ELISA pepsinogen test (PgI/PgII < 3), the latex-agglutination pepsinogen test using a more restrictive criterion (PgI/PgII < 2 and PgI < 30 ng/mL) based on the findings from a previous study [23] and gastrin-17 test (G-17 < 1 pmol/L) to detect the five study outcomes.

Comparison of subject classification between the ELISA and latex-agglutination pepsinogen tests was performed with Pearson correlation coefficient for continuous data and Cohen’s Kappa coefficient and agreement percentage for categorised data.

Individual tests and co-testing performance were explored by means of positivity rates as well as sensitivity and specificity to detect the gastric outcomes described above using the Begg and Greenes method (negative verification fraction of 0.60 and 0.53 and positive verification fraction of 0.77 and 0.79 for pepsinogens and gastrin-17 tests, respectively) to correct for verification bias [24].

Potential adjustment of pepsinogens cut-off point to Latvian population were explored using alternative pepsinogen cut-offs at 0.1 increments in both PgI and PgI/PgII ratio for both ELISA and latex-agglutination pepsinogen tests. Optimal cut-off point values were assessed by highest ROC area, value closest-to-(0,1) corner in the ROC curve, and highest values using the Liu method and Youden Index [25].

All statistical analyses were performed using STATA version 11.0, College station, Texas, USA.

## 4. Results

Biomarkers data were available from 1713 screened subjects balanced on gender (53% females) and age (64% of subjects aged under 55) as per the study design (Total cohort data in Table 1). Among these, 67.9% of subjects were seropositive for *H. pylori* and 15% reported eradication therapy earlier than 1 year from inclusion. Around half of the participants were ever smokers (52%) and 24% reported not drinking in the last 12 months whereas 15% reported an alcohol intake above 10 g per day.

Regarding the study biomarkers, 58.4% (*n* = 1001) met referral criteria for endoscopy (G-17 <1 pmol/L =31.2%, *n* = 535; ELISA PgI/PgII ratio < 3 = 7.7%, *n* = 132; and latex-agglutination: PgI/PgII ratio < 3 and Pgl < 70 ng/mL = 32.5%, *n* = 556). The use of latex-agglutination pepsinogen test with the more restrictive criteria of PgI/PgII ratio < 2 and Pgl < 30 ng/mL would have reduced the endoscopy referral rate to 7.7%.

Evaluation of biomarker performance to detect gastric precancerous lesions was based on the results obtained from the 1045 subjects that underwent upper endoscopy and in whom a full pathology report was available, i.e., 772 (77.1%) of 1001 subjects with at least one of the biomarker results altered and 273 (38.3%) of 712 subjects with all biomarker results normal. The main reasons for subjects with altered biomarkers not undergoing endoscopy were refusal with no explanation (24%), lack of time (23%) and considering it unnecessary (19%). Among all the subjects that underwent upper endoscopy, 44 OLGA ≥ 3, 40 OLGIM ≥ 3, 93 OLGA3 or worse, 53 corpus-restricted atrophy or IM and 108 corpus-restricted atrophy, IM or worse were diagnosed.

**Figure 1:**
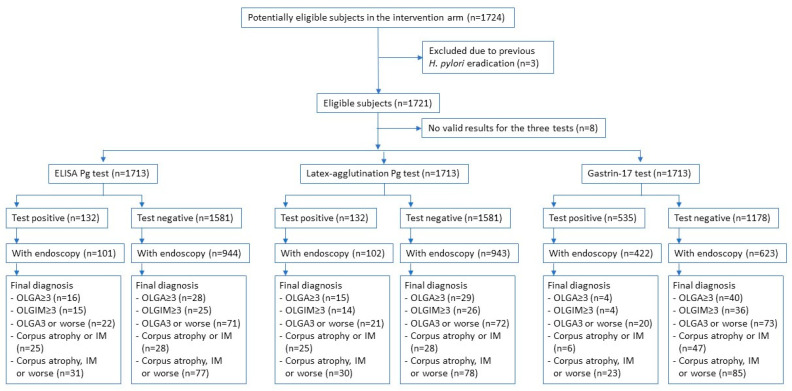
STARD diagram.

The flowchart of study participants and outcomes by biomarker result are detailed in Figure 1. No differences in the descriptive characteristics between the initially screened subjects and those that underwent an endoscopy were observed except for a higher proportion of never smokers in the latter.

Similar performances were observed between the two pepsinogens tests (Table 2); high specificity values (>93%) but low and varying sensitivity values (range: 18.4–31.1%) depending on the outcome explored. Restriction to atrophy or IM lesions in corpus showed better sensitivity (40.5% for both tests), but the study of the composite endpoint including dysplasia resulted in lower sensitivities (23.1 and 23.8% for latex-agglutination and ELISA tests, respectively).

Gastrin-17 alone resulted in lower specificity (67.5% and 67.7%) and sensitivity (6.3% and 6.8%) than pepsinogen testing to detect severe atrophy or severe IM, respectively. As opposed to pepsinogens, the inclusion of dysplasia and cancer improved the sensitivity by 9%, although resulting sensitivity was nevertheless low (15.3%). The performance of gastrin-17 to detect corpus-restricted lesions was not better than that observed in atrophy or IM irrespective of location.

The exclusion of 191 subjects reporting proton pump inhibitors during the prior month to study participation did not significantly change the performance results of pepsinogens and gastrin-17 tests (data not shown).

Due to the similar performance and positivity rates between pepsinogen tests, only the ELISA Pg test was used to explore potential improvements in screening performance in combination with gastrin-17 (). Co-testing with gastrin-17 increased sensitivities to 33–45% although with an important reduction of specificity to 61.1–62% depending on the outcome. This strategy substantially increased the referral rate to 38.6%.

**Figure 2:**
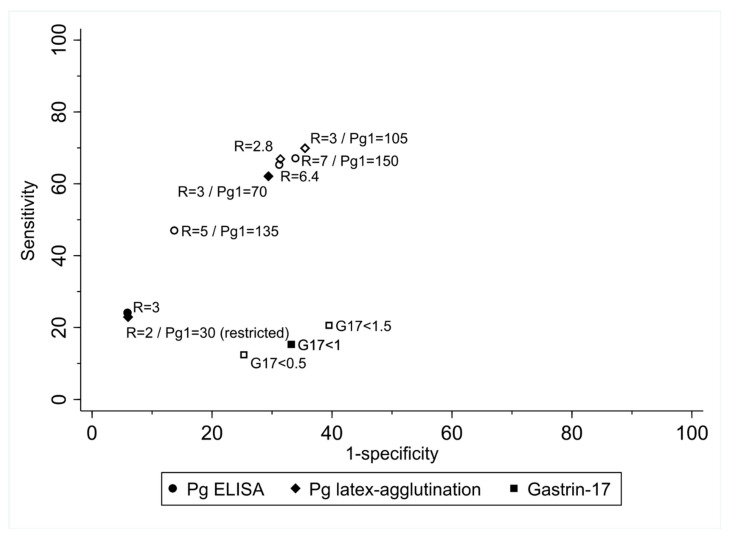
Sensitivity and false positive rate (1-specificity) of ELISA and latex-agglutination pepsinogens tests and gastrin-17 test to detect a combined outcome including corpus atrophy, intestinal metaplasia or worse at standard (solid markers) and best alternative cut-offs with highest ROC area, value closest-to-(0,1) corner in the ROC curve and highest values using the Liu method and Youden Index (blank markers). Abbreviations: Pg = Pepsinogen, R = ratio. Numbers provided are corrected for verification bias using the Begg and Greenes method.

Performance estimates (sensitivity, specificity, predictive values and likelihood ratios) for each test using different cut-offs and the values obtained using the optimal cut-off point methods above-mentioned are provided in Supplementary Materials. Using the atrophy or IM in corpus or worse outcome, there is no outstanding optimal cut-off for any of the tests (Figure 2). A latex-agglutination pepsinogen test with a PgI/PgII ratio cut-off of 2.8 is singled-out by the four explored methods used, but it results in 39.6% positivity to achieve a 71.3% sensitivity and 64.0% specificity.

## 5. Discussion

The potential use of pepsinogens in gastric cancer prevention is still not well-established. Most current European and international guidelines recognise pepsinogen testing as the test of choice for gastric cancer risk stratification, due to its potential to identify gastric precancerous lesions, namely atrophy. The Kyoto Global Consensus emphasizes the role of serology (PgI, Pg II, PgI/PgII ratio and *H. pylori*) in gastric cancer risk stratification [11]. Maastricht V/Florence Consensus supports Pg serology as the most useful non-invasive test to explore the gastric mucosa status (non-atrophic vs atrophic) [10]; a similar recommendation anticipated for the updated version of the Maastricht VI/Florence Consensus [26]). The latest version of the Management of epithelial precancerous conditions and lesions in the stomach guidelines (MAPS II) suggests that low PgI serum levels or/and a low PgI/PgII ratio can be used to identify patients with advanced stages of atrophic gastritis [5]. Some national guidelines, such as those from Brazil [27], also support the use of serology (PgI, PgII, *H. pylori* and gastrin-17) to identify populations at risk of developing gastric cancer. However, the British Society of Gastroenterology guidelines on the diagnosis and management of patients at risk of gastric adenocarcinoma do not recommend the use of biomarkers as a screening tool in areas with a low incidence of gastric adenocarcinoma, such as the UK [28].

Our study shows that pepsinogens test, irrespective of the technology used, have a poor sensitivity (range: 18.4–40.5) but a high specificity (>93%) to detect advanced precancerous lesions. Simultaneous testing for G-17 or use of alternative cut-offs of pepsinogens does not improve accuracy.

Using the ELISA manufacturer’s cut-off value and the latex-agglutination test at the PgI/PgII < 2 and PgI < 30 ng/mL cut-off, both tests showed a similar performance as observed in a previous study [23]. An 8% referral rate for both tests is a likely affordable and acceptable number of subjects undergoing a confirmatory endoscopy. However, sensitivity of these tests substantially varies depending on the outcome used with important reductions in sensitivity when using composite endpoints including more severe outcomes, which might contribute to the perceived futility or refusal to undergo endoscopy among those with an abnormal biomarker result.

A PgI/PgII ratio of 2.8 in latex-agglutination pepsinogen test appeared to be the best cut-off of pepsinogen testing. However, despite an increase in sensitivity to 71.3%, it would result in 4.5 times more subjects undergoing endoscopy, an invasive test, but only 18.6% of which will have disease instead of the 30.5% using the ELISA pepsinogen test at its regular cut-off.

An alternative strategy explored to boost pepsinogen sensitivity is co-testing with other biomarkers. During the recent decade, combined testing of pepsinogens and gastrin-17 has gained significant attention and interest [15]. According to an available meta-analysis, the combination of gastrin-17 and pepsinogen testing increased either the sensitivity or specificity of testing if compared to each of the tests alone [29]. Yet, in our study, this strategy increased the sensitivity by 13.3% up to a detection of 37.1% of lesions requiring surveillance, but the referral rate increased up to 38.4% and almost 40% of the healthy subjects screened would be unnecessarily referred to endoscopy. This limited increase in sensitivity might be explained by the dependence of gastrin-17 levels on several factors, such as ethnicity [30]. Therefore, it is hardly possible to recommend such a testing approach for preventive purposes. Applying the ABC method by Miki et al. [16], i.e., combining serum pepsinogens with *H. pylori* serology would result in a very high referral rate if all the biomarker and *H. pylori* positive cases would get referred given the high *H. pylori* prevalence in our study (68%).

To our knowledge, there are no criteria defined for the minimum values of sensitivity or specificity, and the acceptable referral rates for endoscopy for a screening test implementation to prevent gastric cancer mortality. Overall, screening implementation in any setting depends on factors, among others, such as (i) the costs related to the number of tests and laboratory processing, (ii) tests performance and health care resources (referral rate and subsequent diagnosis, treatment and follow-up management), (iii) burden of disease and (iv) the acceptance and compliance of the population to be screened [31]. Therefore, in the case of gastric cancer prevention, one could assess among others the current use of upper endoscopy as primary gastric cancer screening test, with a moderate-high sensitivity but a reduced participation rate and higher associated costs [32], versus repeated measurement at short intervals of serum biomarkers, cheaper although with a reduced sensitivity, which has proven to be successful for cytology in reducing cervical cancer mortality [33]. Since the specificity of pepsinogen testing was high, a positive result is an accurate indication to perform a confirmatory endoscopy and this could be acceptable if pepsinogen screening tests are performed repeatedly due to their low sensitivity. Furthermore, since other alternatives for this cancer type prevention are available, such as a population-based *screen-and-treat* strategy for *H. pylori*, there is a clear need for a cost-effectiveness modelling for different approaches combined. A recent cost-effectiveness analysis on two potential gastric cancer screening strategies in Portugal showed that upper endoscopy every 5 years was more cost-effective than serum pepsinogens every 2 years [34].

The observed serum biomarkers tests performances, add to the heterogeneous results observed in the individual studies on published meta-analyses [35,36,37]. Its heterogeneity in technology of pepsinogen tests used, the study design (cohort/ case-control) as well as the outcomes explored and their definition (cardia/non-cardia proportion in gastric cancer or grading of atrophy for example) make direct comparison on best cut-off values difficult. However, our study offers a comprehensive assessment of the use of these biomarkers by exploring multiple clinical outcomes, potential strategies of co-testing, different technologies, and assessment of alternative cut-off points. Importantly, the study design resembles that of a population screening program, inviting middle-aged asymptomatic subjects to participate, with a large number of asymptomatic subjects with normal results undergoing endoscopy. We cannot exclude some volunteer bias due to subjects self pre-selection to participate in the study, and asymptomatic subjects to attend endoscopy referral, yet we do not consider this to influence the study results significantly.

## 6. Conclusions

Although non-invasive detection of precancerous stomach lesions remains an attractive strategy to decrease gastric mortality, the estimated sensitivities of stand-alone pepsinogen tests or its combination with gastrin-17 for population-based gastric cancer screenings are not ideal. However, cost-effectiveness analyses are needed to explore its potential for use at repeated short screening intervals.

## Figures and Tables

**Table 1:** Description of study participants; total cohort with valid screening results and those that underwent upper endoscopy.

	Total Cohort		Undergoing Endoscopy	
	No.	%	No.	%
**Gender**				
Female	901	(52.6)	575	(55.0)
Male	812	(47.4)	470	(45.0)
**Age**				
40–54	1088	(63.5)	645	(61.7)
55–65	625	(36.5)	400	(38.3)
**Ever smoker**				
No	891	(52.1)	590	(56.6)
Yes	818	(47.9)	453	(43.4)
**Alcohol intake**				
Non-drinker (0 g/day)	412	(24.1)	252	(24.1)
Regular (<10 g/day)	1045	(61.0)	660	(63.2)
Heavy use (>10 g/day)	255	(14.9)	133	(12.7)
***H. pylori* serology test (≥30 EIU)**				
Negative	550	(32.1)	337	(32.2)
Positive	1163	(67.9)	708	(67.8)
**Screening tests result (endoscopy indication)**				
All biomarkers normal	712	(41.6)	273	(26.1)
1 or more altered	1001	(58.4)	772	(73.9)
**Gastrin-17 test (G-17 < 1 pmol/L)**				
Negative	1178	(68.8)	623	(59.6)
Positive	535	(31.2)	422	(40.4)
**ELISA Pg test (PgI/PgII < 3)**				
Negative	1581	(92.3)	944	(90.3)
Positive	132	(7.7)	101	(9.7)
**Latex-agglutination Pg test (PgI/PgII <3 and Pgl < 70 ng/mL)**				
Negative	1157	(67.5)	626	(59.9)
Positive	556	(32.5)	419	(40.1)
**Latex-agglutination Pg test (PgI/PgII < 2 and PgI < 30 ng/mL)**				
Negative	1581	(92.3)	943	(90.2)
Positive	132	(7.7)	102	(9.8)

ELISA and latex-agglutination with the alternative cut-off restrictive pepsinogens tests showed an almost perfect agreement between them (96.2% agreement) and a high Cohen’s Kappa coefficient of 0.73 in subjects classification (Supplementary Materials).

**Table 2:** Performance of pepsinogen and gastrin-17 tests, individually and co-testing, to detect study outcomes.

	Outcome	TP	FN	FP	TN	Sensitivity (95%CI)	Specificity (95%CI)	PPV (95%CI)	NPV (95%CI)
INDIVIDUAL TESTS									
**ELISA Pg test** **(cut-off: PgI/PgII < 3)**	***OLGA ≥ 3***	21	47	110	1527	30.9 (20.2–43.3)	93.3 (92–94.4)	16 (10.2–23.5)	97 (96–97.8)
	***OLGIM ≥ 3***	19	42	112	1532	31.1 (19.9–44.3)	93.2 (91.9–94.4)	14.5 (9–21.7)	97.3 (96.4–98.1)
	***OLGA3 or worse***	29	118	103	1455	19.7 (13.6–27.1)	93.4 (92–94.6)	22 (15.2–30)	92.5 (91.1–93.8)
	***Corpus atrophy or IM***	32	47	99	1527	40.5 (29.6–52.1)	93.9 (92.6–95)	24.4 (17.3–32.7)	97 (96–97.8)
	***Corpus atrophy, IM or worse***	40	128	91	1445	23.8 (17.6–31)	94.1 (92.8–95.2)	30.5 (22.8–39.2)	91.9 (90.4–93.2)
**Latex-agglutination Pg test (cut-off: PgI/PgII < 2 and PgI < 30 ng/mL)**	***OLGA ≥ 3***	19	48	113	1523	28.4 (18–40.7)	93.1 (91.8–94.3)	14.4 (8.9–21.6)	96.9 (96–97.7)
	***OLGIM ≥ 3***	18	43	114	1528	29.5 (18.5–42.6)	93.1 (91.7–94.2)	13.6 (8.3–20.7)	97.3 (96.3–98)
	***OLGA3 or worse***	27	120	105	1452	18.4 (12.5–25.6)	93.3 (91.9–94.5)	20.5 (13.9–28.3)	92.4 (90.9–93.6)
	***Corpus atrophy or IM***	32	47	100	1525	40.5 (29.6–52.1)	93.8 (92.6–95)	24.2 (17.2–32.5)	97 (96–97.8)
	***Corpus atrophy, IM or worse***	39	130	94	1442	23.1 (17–30.2)	93.9 (92.6–95)	29.3 (21.8–37.8)	91.7 (90.3–93)
**Gastrin-17**	***OLGA ≥ 3***	5	75	529	1100	6.3 (2.1–14)	67.5 (65.2–69.8)	0.9 (0.3–2.2)	93.6 (92.1–94.9)
	***OLGIM ≥ 3***	5	68	529	1108	6.8 (2.3–15.3)	67.7 (65.4–69.9)	0.9 (0.3–2.2)	94.2 (92.7–95.5)
	***OLGA3 or worse***	25	138	509	1038	15.3 (10.2–21.8)	67.1 (64.7–69.4)	4.7 (3.1–6.8)	88.3 (86.3–90)
	***Corpus atrophy or IM***	8	89	527	1087	8.2 (3.6–15.6)	67.3 (65–69.6)	1.5 (0.6–2.9)	92.4 (90.8–93.9)
	***Corpus atrophy, IM or worse***	29	160	505	1015	15.3 (10.5–21.3)	66.8 (64.3–69.1)	5.4 (3.7–7.7)	86.4 (84.3–88.3)
**CO-TESTING**									
**ELISA pg test with Gastrin-17**	***OLGA ≥ 3***	25	48	630	1006	34.2 (23.5–46.3)	61.5 (59.1–63.9)	3.8 (2.5–5.6)	95.4 (94–96.6)
	***OLGIM ≥ 3***	24	42	632	1012	36.4 (24.9–49.1)	61.6 (59.2–63.9)	3.7 (2.4–5.4)	96 (94.7–97.1)
	***OLGA3 or worse***	52	104	604	950	33.3 (26–41.3)	61.1 (58.7–63.6)	7.9 (6–10.3)	90.1 (88.2–91.9)
	***Corpus atrophy or IM***	38	46	618	1008	45.2 (34.3–56.5)	62 (59.6–64.4)	5.8 (4.1–7.9)	95.6 (94.2–96.8)
	***Corpus atrophy, IM or worse***	66	112	590	942	37.1 (30–44.6)	61.5 (59–63.9)	10.1 (7.9–12.6)	89.4 (87.4–91.2)

Abbreviations: TP––True positives, FN––False negatives, FP––False positives, TN––True negatives. Numbers provided are corrected for verification bias using the Begg and Greenes method (negative verification fraction of 0.60 and 0.53 and positive verification fraction of 0.77 and 0.79 for pepsinogens and gastrin-17 tests, respectively). Outcomes: OLGA ≥ 3––Operative link for gastritis assessment (OLGA) Stage 3 or more (severe atrophy); OLGIM ≥ 3––Operative link for gastric intestinal metaplasia (OLGIM) Stage 3 or more (severe IM); OLGA ≥ 3 or worse––composite endpoint including OLGA ≥3, OLGIM ≥ 3, dysplasia and cancer (high risk lesions); Corpus atrophy or IM––Severe atrophy and/or IM restricted to corpus; and Corpus atrophy, IM or worse––Corpus atrophy or IM and dysplasia or cancer irrespective of location.

## Data Availability

Publicly archived datasets are not currently available for the study.

## Supplementary Materials

The following supporting information can be downloaded at: https://www.mdpi.com/article/10.3390/diagnostics12071746/s1, Figure S1. Spearman correlation of Pg I, Pg II and Pg I/II ratio between ELISA and latex-agglutination pepsinogen tests. Figure S2. Concordance between ELISA and latex-agglutination pepsinogen tests results and their performance to detect corpus atrophy, intestinal metaplasia (IM) or worse. Table S1. Alternative cutoff.
