# Supplementary material for: Assessment of Serum Pepsinogens with and without Co-Testing with Gastrin-17 in Gastric Cancer Risk Assessment—Results from the GISTAR Pilot Study
Source: Diagnostics (Basel). 2022 Jul 19;12(7):1746. doi: 10.3390/diagnostics12071746 (PMC9325279; doi:10.3390/diagnostics12071746)
Supplement: Supplementary file 1 [file diagnostics-12-01746-s001.zip › Supp Figure S1 - AGREEMENT.pdf]

Supplemental Figure S1. Spearman correlation of Pg I, Pg II and Pg I/II ratio between ELISA and latex-agglutination pepsinogen tests.

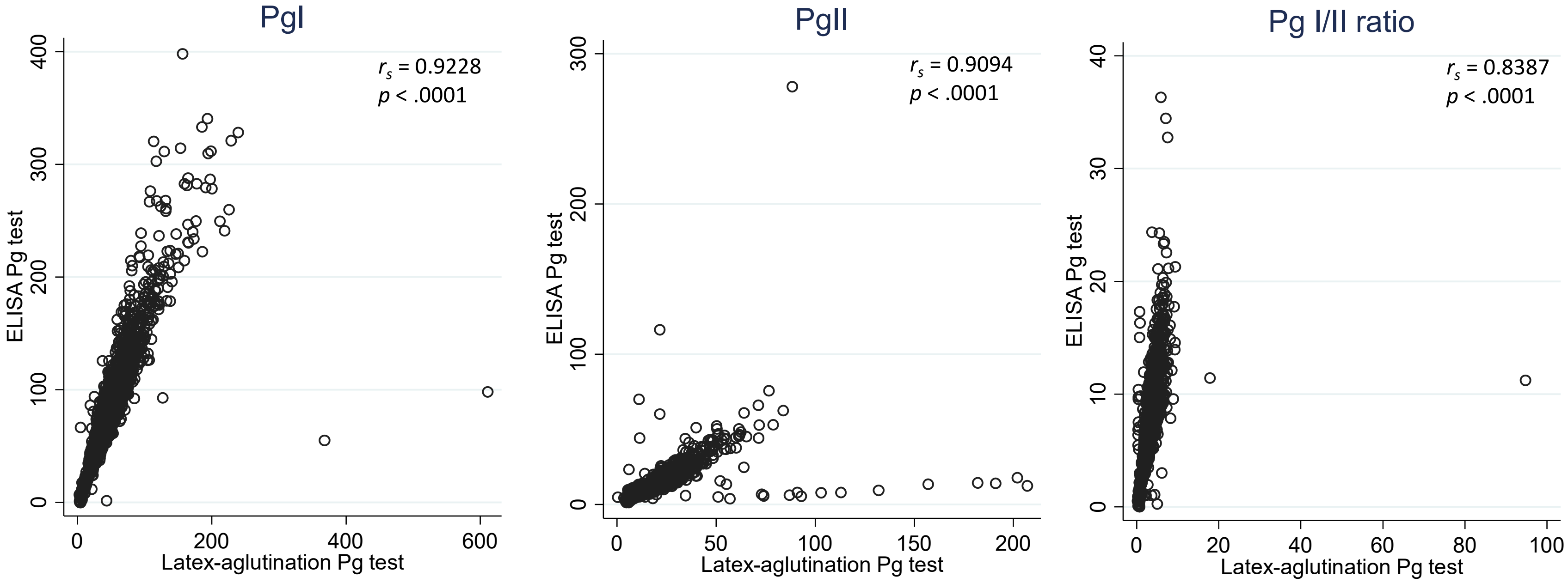


Statistical data has been measured in the 1713 subjects with both pepsinogens data but the graphs do not include seven outlier values of latex-agglutination PgI>1000 and one outlier value of ELISA Pg2>1000.
