# Supplementary material for: Assessment of Serum Pepsinogens with and without Co-Testing with Gastrin-17 in Gastric Cancer Risk Assessment—Results from the GISTAR Pilot Study
Source: Diagnostics (Basel). 2022 Jul 19;12(7):1746. doi: 10.3390/diagnostics12071746 (PMC9325279; doi:10.3390/diagnostics12071746)
Supplement: Supplementary file 1 [file diagnostics-12-01746-s001.zip › Supp Figure S2 - CONCORDANCE.pdf]

Supplemental Figure S2. Concordance between ELISA and latex-agglutination pepsinogen tests results and their performance to detect corpus atrophy, intestinal metaplasia (IM) or worse

ELISA	Latex-agglutination: Neg	Latex-agglutination: Pos
Neg	1548	33
Pos	33	99

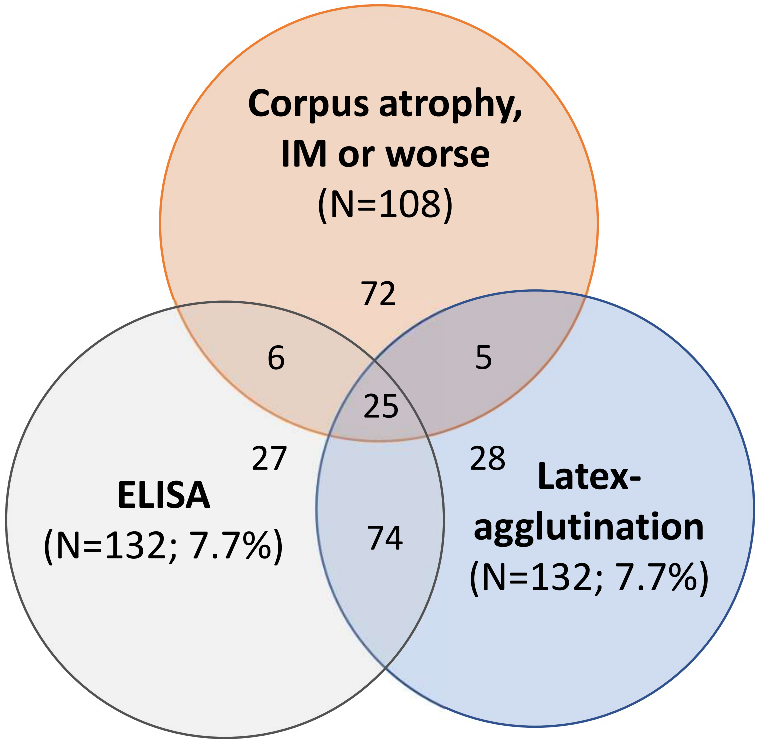

Out of 108 high-risk lesions requiring clinical or surveillance follow-up (severe lesions in corpus, dysplasia or cancer); 23% were detected irrespective of the pepsinogen test used whereas 5 or 6 lesions (5%) were test specific. Double testing could result in an additional 5% sensitivity to detect precancerous lesions but it will increase costs (double processing of samples and an additional 2% referred to endoscopy) to obtain a poor co-testing sensitivity of 33%.
